# Outcome and epilepsy following neonatal stroke in the Italian Registry of Infantile Thrombosis

**DOI:** 10.1007/s00431-026-06912-8

**Published:** 2026-05-07

**Authors:** Stefano Sartori, Margherita Nosadini, Gloria Brigiari, Andrea Francavilla, Thomas Foiadelli, Daniele Veraldi, Agnese Suppiej, Rossana Bagna, Susanna Casellato, Virginia Cao, Laura Baggio, Marta Conti, Daniela Farinasso, Patrizia Accorsi, Duccio Maria Cordelli, Mariella Magarotto, Michela Massoud, Veronica Pegoraro, Maria Federica Pelizza, Paola Saracco, Vittoria Arena, Elisa Ballardini, Alessandra Falcone, Marcella Gaffuri, Diletta Gentile, Alessandro Iodice, Anna Rosati, Samuela Bugin, Elisabetta Chiodin, Veronica Fogliani, Paola Freschi, Maurizio Radicioni, Elena Cavaliere, Irene Toldo, Claudio Ancona, Luca Capato, Jacopo Norberto Pin, Isotta Guidotti, Martina Lombardini, Matteo Luciani, Paolo Simioni, the R. I. T. I. Working group, Francesca Asta, Margherita Baracetti, Roberto Bottino, Mariaelena Cavicchiolo, Silvia Celestino, Gaetano Chirico, Clara Colonna, Placido Currò, Arianna Dagri, Beatrice De Maria, Ambra Fantauzzi, Michela Ada Noris Ferilli, Claudia Gandioli, Sergio Garuccio, Filippo Greco, Patrizia Lo Tartaro Meragliotta, Nicoletta Mainini, Giuliana Marchio’, Valeria Materia, Isabella Mauro, Mariaclaudia Meli, Alessandra Morandi, Giorgio Olzai, Sara Rivellini, Massimo Soffiati, Giuseppina Spanedda, Giorgia Tanzi, Federica Teutonico, Giuseppina Timpani, Marilena Vecchi, Arianna Vincenti, Gianluca Visintin, Elena Pavlidis, Serena Pellegrin, Cesare Zambelloni, Nicoletta Doglioni, Nadia Battajon

**Affiliations:** 1 Department of Women’s and Children’s Health, University of Padua, Clinica Pediatrica, Via Giustiniani 3, 35128 Padua, Italy; 2 Paediatric Neurology and Neurophysiology Unit, Pediatric Division, Department of Women’s and Children’s Health, University-Hospital of Padova, Padua, Italy; 3 Unit of Biostatistics, Epidemiology and Public Health, Department of Cardiac-Thoracic-Vascular Sciences and Public Health, University of Padova, Padua, Italy; 4 Pediatric Clinic, IRCCS Policlinico San Matteo Foundation University of Pavia, Pavia, Italy; 5 Pediatric Unit, Department of Medical Sciences, University Hospital Sant’Anna, University of Ferrara, Ferrara, Italy; 6 Neonatal Intensive Care Unit, University Hospital “Città Della Salute E Della Scienza”, Turin, Italy; 7 Unit of Child Neuropsychiatry, University Hospital of Sassari, Sassari, Italy; 8 Paediatric Division, ULSS2 Marca Trevigiana, Treviso Hospital, Treviso, Italy; 9 Neurology, Epilepsy and Movement Disorders, Full Member of European Reference Network EpiCARE, Bambino Gesù Children’s Hospital, IRCCS, Rome, Italy; 10 Neonatal and Early Childhood Pathology, Regina Margherita Children’s Hospital, City of Health and Science Hospital of Turin, Turin, Italy; 11 Child and Adolescent Neurology and Psychiatry Unit, Children Hospital, ASST Spedali Civili of Brescia, Brescia, Italy; 12 Department of Medical and Surgical Sciences (DIMEC), IRCCS Istituto Delle Scienze Neurologiche Di Bologna, U.O.C. Neuropsichiatria Dell’età Pediatrica, Bologna Italy, Alma Mater Studiorum - University of Bologna, Bologna, Italy; 13 Neonatal Intensive Care Unit, Department of Women’s and Children’s Health, University Hospital of Padova, Padua, Italy; 14 Onco-Hematology, Cell and Gene Therapy and Bone Marrow Transplant Clinic Area, Bambino Gesù Children’s Hospital, IRCCS, Rome, Italy; 15 Unit of Child and Adolescent Neuropsychiatry, Friuli Centrale Health University Authority, Udine, Italy; 16 Pediatric Hematology, Department of Pediatrics, University Hospital Città Della Salute E Della Scienza, Turin, Italy; 17 Neonatal Intensive Care Unit, Sant’Anna Hospital of Ferrara, SantFerrara, Italy; 18 Neonatal Intensive Care Unit GOM Bianchi-Melacrino-Morelli Reggio Calabria, Reggio Calabria, Italy; 19 Department of Surgery, Dentistry, Paediatrics and Gynaecology, Paediatric Clinic, University of Verona, Verona, Italy; 20 Neuroscience Department, Meyer Children’s Hospital IRCCS, University of Florence, Florence, Italy; 21 Child Neuropsychiatry Unit, Pediatric Department S. , Chiara Hospital - APSS, Trento, Italy; 22 U.O.C Pediatria, Ospedale San Bortolo, Vicenza, Italy; 23 Division of Neonatology/NICU, Hospital of Bolzano (SABES-ASDAA), Teaching Hospital of Paracelsus Medical University (PMU), 39100 Bolzano, Italy; 24 Department of Clinical and Experimental Sciences, University of Brescia, Brescia, Italy; 25 Neonatal Intensive Care Unit, Azienda Sanitaria Universitaria Integrata S. Maria Della Misericordia, Udine, Italy; 26 Neonatal Intensive Care Unit and Neonatal Pathology, S. Maria Della Misericordia Hospital, Perugia, Italy; 27 Neonatology and Neonatal Intensive Care Unit, University Hospital of Modena, Modena, Italy; 28 Hemostasis and Thrombosis Center, OncoHematology Unit, Children Hospital Bambino Gesù, Rome, Italy; 29 General Internal Medicine and Thrombotic and Haemorrhagic Unit, University Hospital of Padova, Padua, Italy

**Keywords:** Neonatal arterial ischemic stroke, Neurological outcome, Post-stroke epilepsy, Registry

## Abstract

The perinatal period is a high-risk time for stroke, with possible lifelong effects. We aimed at identifying factors associated with long-term neurological outcomes and post-stroke epilepsy in patients with neonatal arterial ischemic stroke (NAIS). We analyzed patients with NAIS from the Italian Registry of Infantile Thrombosis (RITI). Associations between clinical variables and outcomes (neurological deficits and epilepsy at last follow-up) were evaluated using univariate logistic regression. Among the 181 patients included (56.2% male), seizures were the most common initial symptom (79.4%). Stroke was left-sided in 62.0%, and bilateral in 15.0%; multiple lesions were reported in 32.5%. The middle cerebral artery territory was most frequently involved. One patient had a new infarction during hospitalization, and one died. At follow-up (median 21 months), no further recurrences or deaths occurred; neurological deficits were reported in 38.8%, and post-stroke epilepsy in 12.0% (among these latter, prior acute seizures occurred in 86.7%). At univariate regression, factors significantly associated with long-term neurological deficits were higher maternal age (*p* = 0.031); urgent cesarean (*p* < 0.001); lower gestational age (*p* = 0.033); neurological deficits at discharge (*p* < 0.001); seizures at last follow-up (*p* = 0.008). Factors significantly associated with post-stroke epilepsy were need for acute-phase assisted ventilation (p = 0.001); radiological brainstem involvement (*p* = 0.037); and longer admission duration (*p* = 0.050).

*Conclusion*: Mortality and recurrence after NAIS are rare, but neurological deficits occur in about 40%, and epilepsy in 12%. Perinatal and clinical factors may predict adverse outcomes and epilepsy.

**Table:** 

**What is Known:** • *The perinatal period is a high-risk window for neonatal arterial ischemic stroke (NAIS), with seizures as the most common presenting symptom. Mortality and recurrent strokes after NAIS are rare, but long-term neurological deficits and post-stroke epilepsy are recognized complications.*
**What is New:** • *In this registry-based cohort, specific perinatal and clinical factors—such as higher maternal age, urgent cesarean delivery, lower gestational age, neurological deficits at discharge, and seizures at follow-up—were significantly associated with long-term neurological impairments.* • *Risk factors for post-stroke epilepsy were identified, including need for acute-phase assisted ventilation, radiological brainstem involvement, and longer hospital stays, providing new insights for early prognostication and follow-up strategies.*

**Supplementary Information:**

The online version contains supplementary material available at 10.1007/s00431-026-06912-8.

## Introduction

Perinatal stroke is a diverse but specific group of cerebrovascular diseases that occur between 20 weeks of fetal life and 28 days of postnatal life, with neuroimaging or neuropathological confirmation of cerebral ischemic or hemorrhagic events due to arterial or venous involvement [1, 2]. According to the timing of clinical presentation and diagnosis, perinatal stroke can be further distinguished into acute symptomatic perinatal stroke (when it is diagnosed in the neonatal period, that is within the first 28 days of life, including in preterm infants) and presumed perinatal stroke (when it presents outside the first month of life, most frequently with hemiparetic cerebral palsy, with imaging confirmation of remote stroke that can be presumed to have occurred between 20 weeks of fetal life and 28 days of postnatal life) [1, 3].

Neonatal arterial ischemic stroke (NAIS) is the most common subtype of acute symptomatic perinatal stroke [2], typically presenting in the first 12–72 h of life with focal seizures or other non-focal clinical symptoms, and is confirmed on neuroimaging with a focal area of recent ischemic infarction corresponding to one or more arterial territories [1, 3, 4]. The neonatal age is the most focused life-time period of risk for stroke after the adult period [1, 2], and a relevant proportion of patients with NAIS suffer permanent neurologic morbidity such as hemiparetic cerebral palsy, epilepsy, cognitive, language and behavioural challenges [2]. Nevertheless, several aspects of NAIS are still incompletely understood, such as risk factors and pathogenesis, limiting the development of prevention strategies [4], and research in this field greatly relies on data provided by national and international registry studies [4–7].

In this work, we aimed to report on the Italian cohort of patients with NAIS using the data collected in the Italian Registry of Infantile Thrombosis (RITI), with focus on the identification of potential early factors associated with neurological deficits and post-stroke epilepsy. Moreover, we described risk factors, clinical and radiological presentation.

## Methods

The RITI is a non-interventional retrospective and prospective national Italian registry collecting anonymized data on neonatal and pediatric patients (age 0–18 years) who experienced a systemic or cerebral thrombotic event in the arterial or venous compartment since 2007 (in particular, arterial ischemic stroke [AIS], cerebral sinovenous thrombosis [CSVT]; systemic venous, arterial and intracardiac thrombosis] [7–11].

Data on patients with NAIS were extracted from the RITI registry in September 2023, adhering to the STROBE guidelines. In detail, all patients with NAIS, that is an AIS event occurred between 0 and 28 days of postnatal life, were included. Patients with presumed perinatal AIS or pediatric AIS or neonatal venous stroke or CSVT were excluded.

Acute symptomatic seizures were defined as epileptic seizures occurring within 7 days after NAIS, and post-stroke epilepsy as the occurrence of at least one unprovoked seizure (remote symptomatic seizure) occurring at follow-up [12–14].

We defined neurological deficits as limited or impaired capacity in any of the following areas: muscular tone or movement, cognitive function, emotional-relational regulation, visual or hearing function [15].

The RITI is approved by the institutional Research Ethics Board (Comitato Etico Territoriale #1653P) and this study was conducted in accordance with the principles of the Declaration of Helsinki.

### Statistics

In the descriptive analysis, denominators may vary according to data availability.

For the study of factors associated with neurological deficits and epilepsy at last follow-up, demographic and history data, NAIS risk factors, clinical and radiological data were selected (only the variables with sufficient numerosity for statistical analysis were used). These variables were compared in the two populations of patients who did or did not have neurological deficits at last follow-up and in the two populations of patients who did or did not have epilepsy at last follow-up. The Wilcoxon test was used to calculate the p value for continuous variables and the chi-square test or Fisher’s test (depending on the number) for categorical variables. To evaluate the association between the covariates and the outcome of interest (presence or absence of neurological deficits and of epilepsy at last follow-up), a univariate logistic regression model was implemented and for each covariate the odds ratio (OR), the 95% confidence intervals (95% CI) and the relative *p* value were provided. A *p* value ≤ 0.05 was considered statistically significant.

All statistical analyses were performed with the statistical analysis software R.

## Results

### Cohort description

#### Demographics and family history

We identified 181 patients enrolled in the RITI registry (56.2%, 100/178 males), who presented with NAIS between 2007 and 2023. Ethnicity was Caucasian in 86.9% (146/168), 7.1% (12/168) African, 4.8% (8/168) Asian, and mixed in 1.2% (2/168). Positive family history for thromboembolic events was reported in 13.3% (15/113) of patients.

#### Risk factors

Risk factors reported in our cohort are described below.

#### Maternal, pregnancy, placental and delivery factors

Maternal age at the time of conception was median 32 years (mean 30, standard deviation [SD] 8; data available in 106/181). Medically assisted procreation was reported in 4.8% (8/166) of cases, and twin pregnancy in 3.0% (5/168). Intrauterine growth restriction (IUGR) was reported in 4.5% (7/157).

Maternal infections during pregnancy were reported in 16.1% (23/143), peripartum infections in 10.1% (14/138), positive vaginal swab in 29.3% (34/116). Maternal diabetes was reported in 10.8% (17/157), hypertension in 4.5% (7/157), and substance abuse in 1.3% (2/157).

Placental disorders were described in 7.9% (10/126) (chorioamnionitis in 4.0%, 5/126).

Prolonged rupture of membranes for > 18 h occurred in 10.9% (18/165). Vaginal delivery was reported in 50.0% (86/172) (dystocia in 7.0%, 12/172), and cesarean section in 50.0% (emergent in 31.4%, 54/172). Gestational age at birth was median 39 weeks (mean 38.2 weeks, SD 3.4; data available in 177/181). The Apgar index was < 7 in 35.5% (39/153) of patients at 1 min, and in 10.6% (10/94) at 5 min.

#### Neonatal factors

At least one risk factor specific to the neonatal age was reported in 46.5% (60/129) patients, including: need for resuscitation at birth in 27.1% (35/129), need for mechanical ventilation in 19.4% (25/129), hypoxic ischemic encephalopathy in 11.6% (15/129), hypothermic treatment in 9.3% (12/129), meconium aspiration in 8.5% (11/60), birth trauma in 8.5% (11/60), pulmonary hypertension in 7.8% (10/129), and others.

Additional risk factors reported are detailed in eTable 1.

#### Clinical presentation and treatment

**Fig. 1:**
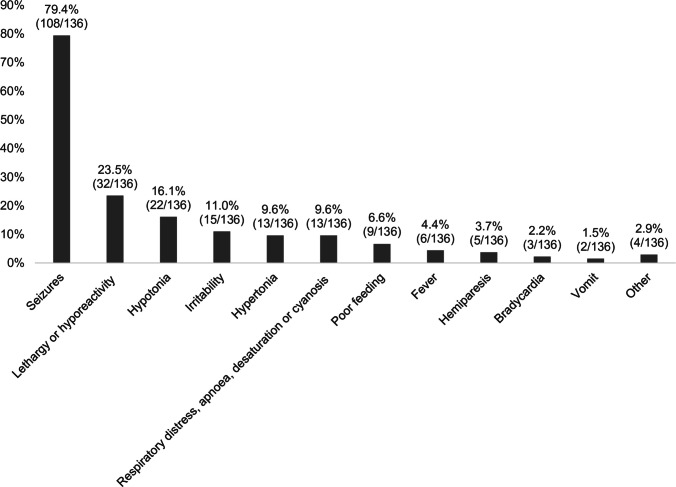
Symptoms at onset of NAIS

In patients with clinical manifestations, most frequent symptoms were acute symptomatic seizures (79.4%, 108/136), lethargy or hyporeactivity (23.5%, 32/136), axial/appendicular hypotonia (16.1%, 22/136), or irritability (11.0%, 15/136) (Fig. 1). Admission to the intensive care unit occurred in 85.5% (153/179). Antithrombotic treatment was used in 16.0% (27/169) (cardiac disorders were reported in 11/27 of these, vasculopathy in 7/27).

#### Radiological findings

Cranial ultrasound was carried out in 77.0% (134/163), brain computed tomography (CT) in 14.4% (24/167), and brain magnetic resonance imaging (MRI) in 93.6% (161/172). Brain MRI showed a new acute or subacute infarction in 80.1% (129/161), a chronic (remote) infarction in 16.8% (27/161), and hemorrhagic conversion in 15.5% (25/161).

A single stroke was reported in 67.5% (110/163), whereas 32.5% (53/163) of patients had multiple strokes. Strokes were left-sided in 62.0% (105/169), right-sided in 23.0% (39/169), and bilateral in 15.0% (25/169). The vascular territory of the middle cerebral artery was most frequently involved. Brain areas affected by the ischemic lesion included the parietal lobe in 60.7% (102/168), the frontal lobe in 53.0% (89/168), the temporal lobe in 47.6% (80/168), the occipital lobe in 19.0% (32.168), the nucleo-capsular region in 21.4% (36/168), the thalamus in 19.6% (33/168), the basal ganglia in 10.1% (17/168), the cerebellum in 3.6% (6/168), and the brainstem in 3.6% (6/168).

#### Outcome

Admission duration was median 19 days (IQR 12, 28), mean 25 days (SD 21) (data available in 152/181), and median age at last follow-up was 1.8 years (IQR 1.1–3.8) (data available in 125/181).

One patient died during admission after the first stroke (0.6%, 1/166), but no further deaths were reported at follow-up.

At repeat brain MRI during admission (done in 22.1%, 40/181 of patients), hemorrhagic transformation was described in 7.5% (3/40), and a new infarction was reported in 2.5% (1/40) of cases, but no further AIS recurrences were reported at last follow-up. Whereas, extracerebral thrombosis was reported in 0.6% (1/181) at discharge, and in 1.6% (2/125) of patients at last follow-up.

**Fig. 2:**
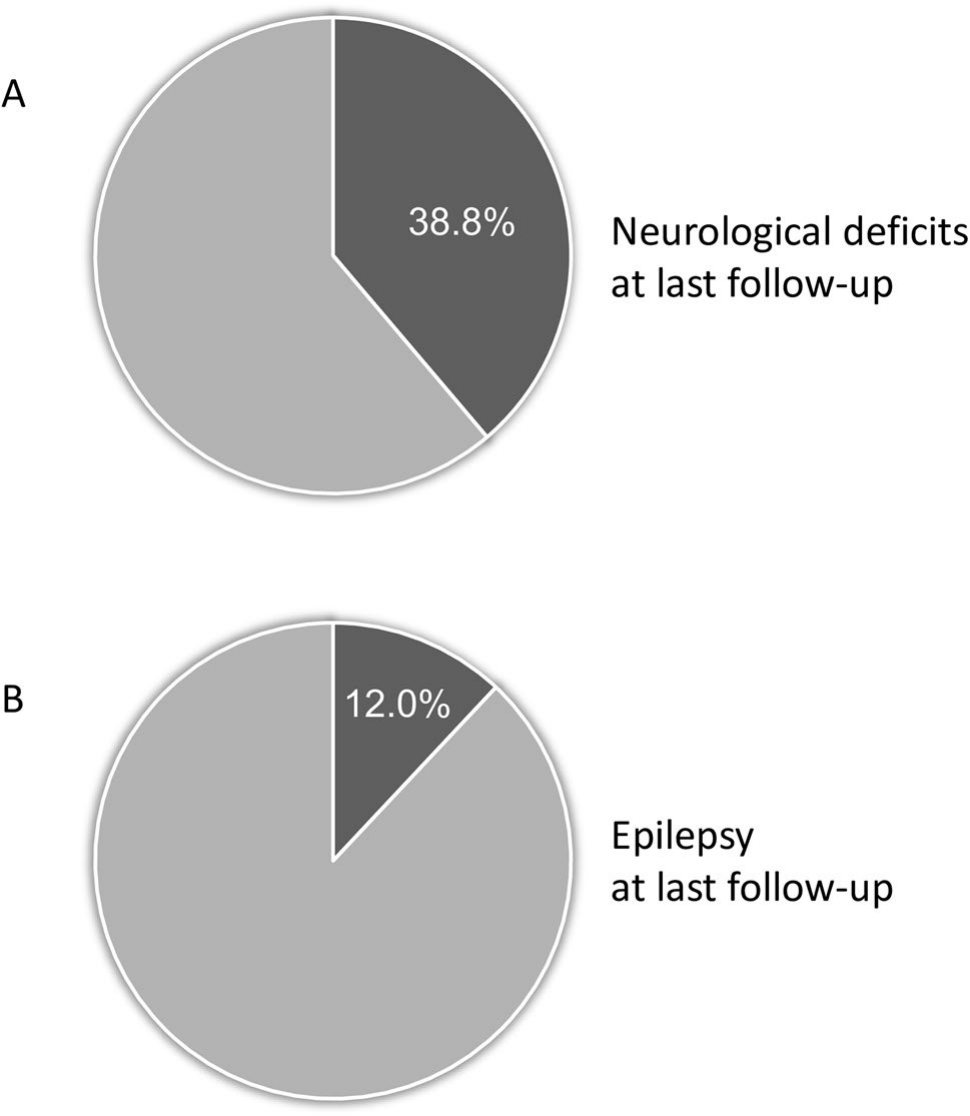
Proportion of patients with NAIS with neurological deficits (**A**) and epilepsy (**B**) at last follow-up

Neurological deficits at discharge were reported in 42.9% (69/161), most frequently abnormal muscular tone (56.5%, 39/69) or motor deficits (50.7%, 35/69), followed by relational (14.5%, 10/69), visual (11.6%, 8/69), cognitive (4.3%, 3/69), or hearing (2.9%, 2.69) deficits. At last follow-up, neurological deficits were reported in 38.8% (47/121) of cases (Fig. 2A). In a subset of these patients with neurological deficits at follow-up and available data, the Pediatric Stroke Outcome Measure (PSOM) was 0.5 in 30.4% (7/23), 1–2 in 52% (12/23), and > 2 in 17.4% (4/23). Seizures at follow-up (post-stroke epilepsy) occurred in 12.0% (15/125) of patients (Fig. 2B); of these, 86.7% (13/15) had already had seizures in the acute phase (at presentation or during admission after stroke).

### Factors associated with neurological deficits and epilepsy at last follow-up

#### Outcome at last follow-up

At univariate regression analysis (Table 1), factors significantly associated with neurological deficits at last follow-up included:

- Maternal age at the time of conception ≥ 32 years: OR 3.21, IC 95% 1.14–9.76, *p* value 0.031;
- Urgent cesarean section: OR 6.52, IC 95% 2.53–17.9, *p* value < 0.001;
- Lower gestational age: OR 0.88, IC 95% 0.78–0.99, *p* value 0.033 (each week of lower gestational age was associated with 14% higher probability of neurological deficits at last follow-up);
- Neurological deficits at discharge: OR 6.98, IC 95% 3.14–16.3, *p* value < 0.001;
- Epileptic seizures at last follow-up: OR 5.28, IC 95% 1.64–20.3, *p* value 0.008.

**Table 1:** Factors statistically associated to the presence of neurological deficits at last follow-up

Variables	Univariate regression			
	N	OR^*3*^	95% CI^*3*^	*p*-value
**Demographics**				
Gender	126			
Female		—	—	
Male		0.54	0.26, 1.12	0.10
Delivery	122			
Vaginal eutocic		—	—	
Emergency cesarean section		6.52	2.53, 17.9	** < 0.001**
Gestational age	126	0.88	0.78, 0.99	**0.033**
Family history of thromboembolism	86			
No		—	—	
Yes		0.75	0.15, 2.85	0.7
Maternal age at conception	72			
< 32		—	—	
> = 32		3.21	1.14, 9.76	**0.031**
**Risk factors**				
**Maternal and placental factors**				
Vaginal swab	89			
Negative		—	—	
Positive		0.63	0.19, 1.84	0.4
Maternal infections in pregnancy	103			
No		—	—	
Yes		1.31	0.37, 4.30	0.7
Peripartum maternal infections	102			
No		—	—	
Yes		0.91	0.18, 3.69	0.9
Other pregnancy conditions	116			
No		—	—	
Yes		1.16	0.48, 2.75	0.7
Placental disorders	84			
No		—	—	
Yes		1.79	0.31, 10.2	0.5
**Fetal and neonatal factors**				
Cardiopathy	126	1.86	0.86, 4.01	0.11
Infection	126	1.15	0.44, 2.87	0.8
Thrombophilia screening	74			
Negative		—	—	
Positive		0.87	0.34, 2.22	0.8
Central vessel catheterisation	124	0.82	0.31, 2.05	0.7
Resuscitation at birth	109	1.21	0.48, 2.99	0.7
Hypoxic-ischaemic encephalopathy	109	0.63	0.19, 1.88	0.4
Hypothermia	109	0.29	0.06, 0.98	0.068
Meconium aspiration	109	0.35	0.02, 2.50	0.4
Birth trauma	109	0.48	0.02, 3.90	0.5
Pulmonary hypertension	109	0.73	0.10, 3.90	0.7
Mechanical ventilation	109	2.76	0.99, 8.16	0.055
**Clinical data**				
Symptoms at presentation	124			
No		—	—	
Yes		0.64	0.21, 1.94	0.4
Need for intensive care unit	124			
No		—	—	
Yes		0.53	0.20, 1.40	0.2
Fever	111	0.54	0.03, 4.35	0.6
Poor sucking	111	0.54	0.03, 4.35	0.6
Irritability	111	2.20	0.55, 9.36	0.3
Lethargy/drowsiness/hyporeactivity	111	0.92	0.34, 2.40	0.9
Epileptic seizures	111	0.59	0.25, 1.36	0.2
Hypotonia	111	0.71	0.21, 2.13	0.6
Hypertonia	111	0.19	0.01, 1.07	0.12
**Neuroimaging**				
Hemorrhagic component	126	1.19	0.43, 3.14	0.7
Side	120			
Bilateral		—	—	
Unilateral		0.41	0.14, 1.19	0.10
Fontal lobe	120	1.81	0.86, 3.87	0.12
Temporal lobe	120	1.65	0.79, 3.51	0.2
Parietal lobe	120	1.59	0.75, 3.44	0.2
Occipital lobe	120	0.49	0.16, 1.28	0.2
Nucleo capsular region	120	1.77	0.74, 4.24	0.2
Thalamus	120	1.58	0.65, 3.83	0.3
Cerebellum	120	1.70	0.20, 14.6	0.6
Brainstem	120	29,346,301	0.00, NA	> 0.9
Basal ganglia	120	1.77	0.52, 6.02	0.4
Number of infarcts	120			
Multiple		—	—	
Single		0.90	0.41, 1.98	0.8
Presence of haemorrhage	27			
Yes		—	—	
No		2.29	0.49, 11.9	0.3
**Other**				
EEG	107			
Normal		—	—	
Abnormal		0.71	0.27, 1.93	0.5
Localised obstruction	126	1.46	0.63, 3.36	0.4
Embolism	126	0.77	0.25, 2.12	0.6
Systemic hypoperfusion	126	1.35	0.42, 4.16	0.6
**Outcome at discharge**				
Days of admission	114			
< 19		—	—	
> = 19		1.96	0.91, 4.31	0.087
Epileptic seizures during admission	122			
No		—	—	
Yes		0.76	0.33, 1.66	0.5
Chronic antiseizure medications at discharge	39			
No		—	—	
Yes		2.44	0.58, 12.9	0.2
Neurological deficits at discharge	121			
No		—	—	
Yes		6.98	3.14, 16.3	** < 0.001**
Interaction/relational deficits	126	11.8	1.93, 228	**0.024**
Movement deficits	126	5.07	2.09, 13.1	** < 0.001**
Muscular tone deficits	126	2.51	1.09, 5.88	**0.031**
**Clinical data at last follow-up**				
Epileptic seizures at follow-up	126			
No		—	—	
Yes		5.28	1.64, 20.3	**0.008**

#### Post-stroke epilepsy at last follow-up

At univariate regression analysis (Table 2), factors significantly associated with seizures at last follow-up (post-stroke epilepsy) included:

- Need for assisted ventilation in the acute phase: OR 10, IC 95% 2.51–44.3, *p* value 0.001;
- Brainstem involvement at MRI: OR 7.63, IC 95% 0.92–52.3, *p* value 0.037;
- Admission duration ≥ 19 days: OR 8.32, IC 95% 1.45–157, *p* value 0.050.

**Table 2:** Factors statistically associated to the presence of epilepsy at last follow-up

Variables	N	OR^***1***^	95% CI^*a*^	*p*-value
**Demographics**				
Gender	119			
Female		—	—	
Male		2.87	0.78, 13.6	0.13
Delivery	115			
Vaginal eutocic		—	—	
Emergency cesarean section		1.20	0.22, 5.81	0.8
Gestational age	119	0.88	0.76, 1.02	0.068
Family history of thromboembolism	79	1.26	0.06, 8.96	0.8
No	65			
Yes		—	—	
Maternal age at conception		0.39	0.05, 2.18	0.3
**Risk factors**				
**Maternal and placental factors**				
Vaginal swab	82			
Negative		—	—	
Positive		0.41	0.02, 2.54	0.4
Other disorders in pregnancy	109	0.67	0.10, 2.87	0.6
Placental disorders	84	2.03	0.10, 15.2	0.5
**Fetal and neonatal factors**				
Cardiopathy	119	1.67	0.45, 5.90	0.4
Infection	119	2.74	0.55, 10.9	0.2
Resuscitation at birth	102	1.36	0.28, 5.37	0.7
Hypoxic-ischaemic encephalopathy	102	1.17	0.06, 7.51	0.9
Meconium aspiration	102	2.44	0.12, 18.9	0.4
Pulmonary hypertension	102	1.93	0.10, 13.9	0.6
Mechanical ventilation	102	10.0	2.51, 44.3	**0.001**
**Clinical data**				
Symptoms at presentation	117	0.63	0.14, 4.42	0.6
Intensive care unit admission	117	0.31	0.08, 1.30	0.087
Fever	108	3.13	0.15, 27.3	0.3
Lethargy/drowsiness/ipohyporeactivity	108	0.36	0.02, 2.05	0.3
Epileptic seizures	108	1.14	0.30, 5.47	0.9
Hypotonia	108	0.55	0.03, 3.18	0.6
**Neuroimaging**				
Haemorrhagic component	119	2.01	0.41, 7.77	0.3
Side	117			
Bilateral		—	—	
Unilateral		0.68	0.16, 4.79	0.6
Fontal lobe	117	1.11	0.32, 4.07	0.9
Temporal lobe	117	1.29	0.37, 4.74	0.7
Parietal lobe	117	1.97	0.54, 9.36	0.3
Occipital lobe	117	1.02	0.15, 4.36	> 0.9
Nucleo capsular region	117	3.18	0.85, 11.5	0.075
Thalamus	117	1.35	0.28, 5.11	0.7
Brainstem	117	7.63	0.92, 52.3	**0.037**
Basal ganglia	117	2.13	0.30, 9.84	0.4
Number of infarcts	117			
Multiple		—	—	
Single		0.90	0.25, 3.62	0.9
**Other**				
EEG	104			
Normal		—	—	
Abnormal		0.88	0.19, 6.19	0.9
Localised obstruction	119	0.74	0.11, 3.10	0.7
Embolism	119	1.19	0.17, 5.14	0.8
Systemic hypoperfusion	119	1.78	0.25, 7.99	0.5
**Outcome at discharge**				
Length of hospitalization (days)	111			
< 19		—	—	
> = 19		8.32	1.45, 157	**0.050**
Epileptic seizures during hospitalization	119	2.73	0.77, 10.1	0.12
Chronic antiseizure medications at discharge	39	1.14	0.19, 9.13	0.9
Neurological deficits at discharge	118			
No		—	—	
Yes		2.32	0.66, 9.30	0.2
Interaction/relational deficits	119	4.58	0.60, 24.9	0.093
Moviment deficits	119	1.31	0.27, 4.94	0.7
Muscular tone deficits	119	1.12	0.23, 4.21	0.9
**Clinical data at last follow-up**				
Neurological deficits at follow-up	119	3.10	0.88, 12.4	0.086

^*a*^*OR* Odds Ratio, *CI* Confidence Interval

## Discussion

In this work, we have reported data on 181 patients with NAIS enrolled in the Italian RITI registry, with focus on outcome, on the identification of early features associated with long-term neurological deficits and post-stroke epilepsy, and on the description of risk factors for NAIS.

Mortality after NAIS is low (only one patient in our cohort, and about 0.0%–2.5% in the literature) [4, 6, 16, 17]. Similarly, recurrence of AIS is very rare after NAIS (only one patient in our cohort), possibly reflecting the peculiar maternal–fetal interplay in the pathogenesis of NAIS.

Although, neurological deficits are reported in a significant proportion of patients — around 40% in our cohort (Fig. 2A), and up to 60% in the literature [6, 16, 18]. Notably, neurological deficits have been reported to “emerge” during follow-up [6], therefore it could be hypothesized that, with a longer follow-up, a higher range of neurological deficits would be identifiable and become manifest in these patients. Indeed, it is possible that more subtle impairments may not be visible in young children due to the limited duration of follow-up.

Muscular tone and movement were by far the most frequently affected domains in our cohort, and cerebral palsy is described in the literature in about a third of patients after NAIS, besides other deficits including epilepsy, cognition, language, behavior, mood, and vision impairments [6, 18, 19].

Factors associated with long-term neurological outcome have not been definitely clarified [20]. In our cohort, factors significantly associated with neurological deficits at last follow-up included maternal age at the time of conception ≥ 32 years, urgent cesarean section, lower gestational age, neurological deficits at discharge, epileptic seizures at last follow-up. Of these, the role of urgent cesarean section may be an expression of the NAIS itself, or of an underlying phenomenon that may have facilitated the stroke. The rate of neurological deficits at the time of discharge was similar to that at last follow-up in our cohort and, predictably [21], was associated with persistence of deficits at last follow-up; therefore, our results show that the clinical examination at discharge may already serve as an important tool for counseling the family, especially if combined with other indicators. As regards seizures, post-stroke epilepsy has been associated with poor outcome in the literature [22], possibly reflecting a more severe ischemic lesion and the detrimental effect of seizures on quality of life. Other factors variably reported to be associated with long-term neurological deficits in the literature include neonatal seizures [21], size of the lesion [3, 16], stroke location [3, 18, 23, 24], bilateral lesions [16]. Some of these factors, such as stroke size, could not be precisely analyzed because this information is not collected in the R.I.T.I. registry questionnaire, representing a limitation of its design. As a surrogate measure, we examined the association of bilateral stroke and of multiple strokes with neurological outcome (see Table 1); however, these associations did not reach statistical significance.

Post-stroke epilepsy after NAIS was reported in about 12% in our cohort (Fig. 2B) and in 10–30% in the literature [14, 17, 18, 22, 25–27]. In our cohort, factors significantly associated with post-stroke epilepsy included need for assisted ventilation in the acute phase, brainstem involvement at MRI, and admission duration ≥ 19 days. These may represent indirect indicators of severe disease. Furthermore, regarding brainstem injury, a potential prognostic role of cerebral peduncle involvement for future epilepsy was previously described in the literature [18]. This has been attributed to a pre-Wallerian degeneration process affecting the descending axons from injured neuronal cell bodies within the infarcted areas, which represents a sign of significant ischemic cortical injury that may precede the development of an epileptogenic process. Notably and similarly to other literature data [22, 25, 27], most of the patients with post-stroke epilepsy had neonatal seizures in the acute phase (86.7%, 13/15), although this did not reach statistical significance in our cohort. Other factors reported in the literature in association with post-stroke epilepsy include lesion size [14, 27] and location [18], involvement of the right middle cerebral artery and multiple territories [26], and bilateral lesions [18].

NAIS etiology is considered to be multifactorial, although the specific cause remains unclear in a considerable proportion of cases [16, 20, 28]. Moreover, the risk factors associated with NAIS differ to those described in the adult and pediatric age, and are generally grouped into antenatal (maternal, pregnancy and placental factors), perinatal (birth/delivery complications), and neonatal factors [16, 18]. These factors are the expression of the unique, complex and evolving interplay between the maternal, placental and fetal systems, accompanying the newborn from the in-utero stage to the extrauterine life, and the extent of the causative role they may individually cover may be difficult to establish. For example, age-specific neonatal factors and comorbidities were frequently reported (46.5%, 60/129) in our cohort (most frequently need for resuscitation or mechanical ventilation at birth, hypoxic ischemic encephalopathy, hypothermia, meconium aspiration, birth trauma, etc.). These may have played a role in the pathogenesis of the NAIS or, in some cases, may have been a complication related to the NAIS itself or to an underlying condition. On the other hand, risk factors that play a major role in pediatric AIS, such as vasculopathy and cardiopathy, were only infrequently described in our cohort of patients with NAIS, supporting a different pathogenesis in these age groups (eTable 1). Although extremely complex, the accurate identification of risk factors associated with NAIS may serve as the base both to understand NAIS pathogenesis further, and potentially for the creation of integrated risk prediction model to assess the probability of NAIS in neonates [29], shedding some light into the future possibility of putting into place more effective and personalized prevention strategies.

The unique features of NAIS, compared to pediatric AIS, are also confirmed in the acute clinical symptoms: indeed, NAIS is characterized by a remarkably higher rate of presentation with seizures (88% in neonates vs 37% in children), and by a considerably lower rate of focal neurological deficits (12% vs 77%) [6], as is also reflected by our registry.

While antithrombotic treatment is generally not recommended in NAIS, it can be considered in specific situations [30, 31], and it was reported in a proportion of our cohort (16.0%, 27/169); among these cardiac disorders and vasculopathy were described in a subset, besides other possible risk factors, and may have played a role in the decision to treat. Moreover, since the centers participating in the RITI registry are frequently third-level referral centers managing highly complex patients, the registry questionnaire may not have fully captured the complexity of these cases or the need for individualized treatment in certain situations. Finally, it should also be considered that the RITI registry reflects the real-world clinical practice of a large number of Italian centers and may, in some instances, have captured suboptimal adherence to guideline recommendations, particularly in light of the long enrolment period.

### Limitations

The main limitations of our work include those inherent to the RITI registry [7], especially as regards the registry structure (i.e. some specific information not collected in the registry questionnaire, such stroke size, pre-Wallerian degeneration, or involvement of the posterior limb of the internal capsule), the incomplete data availability and the limited follow-up duration and information. In particular, a significant drop was observed in the number of patients with available data at follow-up [7], although this is similar to other literature cohorts [16, 17]. The overall heterogeneity in data availability stems from the fact that, although a large amount of information can be collected in the RITI registry, only a very limited dataset is mandatory for the enrollment of a given patient. This approach was designed to maximize patient inclusion, even in cases where complete data are not available [7]. Information about the specific neonatal seizure type and timing of occurrence was lacking. During follow-up, a standardized scale for outcome assessment (such as PSOM) was used in only a minority of cases, making it more difficult to compare the outcome results. Similarly, other standardized scales were not captured in the R.I.T.I. questionnaire. The design of our study did not allow to compare risk factors for NAIS between our NAIS cohort and healthy controls, so a descriptive analysis was carried out with comparison to the existing literature, especially in view of the comprehensive and detailed data provided by the RITI registry.

## Conclusions

Despite these limitations, the RITI registry contributes to the literature with a sizeable cohort of patients with NAIS, with further insight into the characteristics of associated factors, and the identification of early factors that may be associated with long-term neurological outcome and post-stroke epilepsy.

## Supplementary Information

Below is the link to the electronic supplementary material.

ESM1 (DOCX 21.3 KB)

## Data Availability

No datasets were generated or analysed during the current study.

## Abbreviations

AIS: Arterial ischemic stroke
CSVT: Cerebral sinovenous thrombosis
CT: Computed tomography
MRI: Magnetic resonance imaging
NAIS: Neonatal arterial ischemic stroke
PSOM: Pediatric stroke outcome measure
RITI: Italian Registry of Infantile Thrombosis
