# Supplementary material for: Outcome and epilepsy following neonatal stroke in the Italian Registry of Infantile Thrombosis
Source: Eur J Pediatr. 2026 May 7;185(6):352. doi: 10.1007/s00431-026-06912-8 (PMC13149617; doi:10.1007/s00431-026-06912-8)
Supplement: Supplementary file 1 — (DOCX 21.3 KB) [file 431_2026_6912_MOESM1_ESM.docx]

**eTable 1: Main risk factors associated with NAIS: maternal pregnancy and placental factors (A), delivery factors (B), neonatal factors (C), other factors (D)**

| 1. **A. Maternal, pregnancy, and placental factors** |  |
| --- | --- |
| Maternal age at the time of conception | Median 32 years (mean 30, SD 8; d.a. 106/181) |
| Medically assisted procreation | 4.8% (8/166) |
| Twin pregnancy | 3.0% (5/168) |
| Maternal infections during pregnancy | 16.1% (23/143) |
| Maternal peripartum infections | 10.1% (14/138) |
| Positive vaginal swab | 29.3% (34/116) |
| Other maternal conditions and risk factors during pregnancy | 24.8% (39/157) |
| Gestational diabetes | 10.8% (17/157) |
| Hypertension | 4.5% (7/157) |
| IUGR | 4.5% (7/157) |
| Autoimmune thyroid disease | 2.5% (4/157) |
| Other thyroid disorders | 4.5% (7/157) |
| Substance abuse | 1.3% (2/157) |
| Systemic lupus erythematosus / Antiphospholipid syndrome / Smoke / Antiseizure medications | 2.5% (4/157) (1/157 each) |
| Placental disorders | 7.9% (10/126) |
| Fetal thrombotic vasculopathy | 3.2% (4/126) |
| Histological chorioamnionitis | 2.4% (3/126) |
| Clinical chorioamnionitis | 1.6% (2/126) |
| Abruptio placentae | 0.8% (1/126) |
| **B. Delivery factors** |  |
| Prolonged rupture of membranes for >18 hours | 10.9% (18/165) |
| Eutocic vaginal delivery | 43.0% (74/172) |
| Dystocic vaginal delivery | 7.0% (12/172) |
| Elective caesarean section | 18.6% (32/172) |
| Emergency caesarean section | 31.4% (54/172) |
| 5-minute Apgar index <7 | 10.6% (10/94) |
| 5-minute Apgar index 7-10 | 89.4% (84/94) |
| **C. Neonatal factors** |  |
| Risk factors specific to the neonatal age | 46.5% (60/129) |
| Need for resuscitation at birth | 27.1% (35/129) |
| Need for assisted ventilation | 19.4% (25/129) |
| Hypoxic ischemic encephalopathy | 11.6% (15/129) |
| Hypothermic treatment | 9.3% (12/129) |
| Meconium aspiration | 8.5% (11/60) |
| Birth trauma | 8.5% (11/60) |
| Pulmonary hypertension | 7.8% (10/129) |
| Persistent ductus arteriosus | 3.1% (4/129) |
| Systemic hypertension | 2.3% (3/129) |
| Pulmonary bronchodysplasia | 2.3% (3/129) |
| Organ malformations, erythroblastic disease or retinopathy of the prematurity | 15.5% (2/129) |
| Metabolic disorder with onset in neonatal age | 7.8% (1/129) |
|  |  |
| **D. Other factors** |  |
| **Thrombophilia (≥1 abnormal)** | 46.4% (45/97) |
| MTHFR C677T polymorphism | 20.6% (20/97) |
| Factor V Leiden | 5.2% (5/97) |
| PT G20210A mutation | 4.1% (4/97) |
| MTHFR A1298C polymorphism / Anti-phospholipid syndrome | 6.2% (6/97) (3/97 each) |
| Hereditary protein C deficiency / Hereditary protein S deficiency / Hereditary antithrombin deficiency / Double heterozygosity, double defect or combined defect | 4.1% (4/97) (1/97 each) |
| **Coagulation abnormalities (≥1 abnormal)** | 18.6% (18/97) |
| Reduction of factor IX / Reduction of factor XI | 5.2% (5/97) / 5.2% (5/97) |
| Reduction of antithrombin levels | 3.1% (3/97) |
| Reduction of factor II / Reduction of factor X | 2.1% (2/97) / 2.1% (2/97) |
| Shortening of prothrombin time (Pt) / Longer activated thromboplastin time (aPTT) / Reduction of factor VII / Increased factor VIII | 4.1% (4/97) (1/97 each) |
| **Cardiac disorders** | 32.4% (57/176) |
| Isolated patent foramen ovale | 22.7% (40/176) |
| Congenital cardiopathy | 7.4% (13/176) |
| Isolated patent ductus arteriosus | 5.7% (10/176) |
| Heart failure / Pulmonary hypertension | 3.4% (6/176) (3/176 each) |
| Arrhythmia / Tricuspid insufficiency | 2.3% (4/176) (2/176 each) |
| Cardiomyopathy | 0.6% (1/76) |
| **Vasculopathy** | 7.5% (13/173) |
| Vascular malformations or dissections | 6.4% (11/173) |
| Other vasculopathies (Vasculopathies due to infections, neoplasms or medications / Primary isolated central nervous system vasculitis / Vasculopathy due to connective tissue disorders) | 1.8% (3/171) (1/171 each) |
| **Infections** | 18.2% (32/176) |
| Sepsis | 9.7% (17/176) |
| Febrile events | 4.0% (7/176) |
| CNS infections | 1.7% (3/176) |
| Urinary tract infections | 1.7% (3/176) |
| Pulmonary infections | 0.6% (1/176) |
| Other infections | 1.7% (3/176) |
| **Non-neoplastic hematologic conditions** | 13.5% (23/171) |
| Anemia | 7.0% (12/171) |
| Non-0 blood type | 5.8% (10/171) |
| Others | 5.3% (9/171) |
| **Hypoglycemia** | 8.0% (11/138) |
| **Metabolic disorders** | 3.4% (6/174) |
| **Liver disorders** | 1.7% (2/173) |
| **Central vessel catheterization** | 20.0% (35/171) |
| **Surgery / Extracorporeal membrane oxygenation** | 2.3% (4/173) (2/173 each) |
| **Cardiac catheterization / Dyalisis** | 1.2% (2/172) (1/172 each) |

It should be noted that a considerable proportion of cases categorized under “cardiac disorders” consisted of isolated patent foramen ovale, a finding that is frequently physiological in the neonatal period.
